# Epidemiology of Pediatric NMOSD in Germany and Austria

**DOI:** 10.3389/fneur.2020.00415

**Published:** 2020-05-15

**Authors:** Christian Lechner, Markus Breu, Eva-Maria Wendel, Barbara Kornek, Kathrin Schanda, Matthias Baumann, Markus Reindl, Kevin Rostásy

**Affiliations:** ^1^Division of Pediatric Neurology, Department of Pediatrics I, Medical University of Innsbruck, Innsbruck, Austria; ^2^Department of Pediatrics and Adolescent Medicine, Medical University of Vienna, Vienna, Austria; ^3^Department of Pediatrics, Olgahospital Stuttgart, Stuttgart, Germany; ^4^Department of Neurology, Medical University of Vienna, Vienna, Austria; ^5^Department of Neurology, Medical University of Innsbruck, Innsbruck, Austria; ^6^Department of Pediatric Neurology, Children's Hospital Datteln, University Witten/Herdecke, Datteln, Germany

**Keywords:** NMOSD, AQP4-antibodies, MOG-antibodies, transverse myelitis, optic neuritis, brainstem syndrome

## Abstract

**Background:** Neuromyelitis optica spectrum disorders (NMOSD) are severe inflammatory demyelinating disorders of the central nervous system mainly characterized by recurrent episodes of uni- or bilateral optic neuritis (ON), transverse myelitis (TM) and brainstem syndromes (BS). The majority of adult patients has serum antibodies directed against the water channel protein aquaporin 4 (AQP4-abs). In pediatric patients, AQP4-abs are less, while antibodies against myelin oligodendrocyte glycoprotein (MOG-abs) are more frequently detectable than in adults. Some children with NMOSD have neither AQP4- nor MOG-ab (double-seronegative).

**Objective:** Evaluation of epidemiological data regarding incidence and prevalence of pediatric NMOSD in Germany and Austria.

**Methods:** We recruited pediatric NMOSD patients between 1 March 2017 and 28 February 2019 with five different tools: (1) ESPED (Surveillance Unit for Rare Pediatric Disorders in Germany), (2) ESNEK (Surveillance for Rare Neurological Disorders during Childhood), (3) pediatric neurology working group within the Austrian Society of Pediatrics and Adolescent Medicine, (4) BIOMARKER Study and (5) NEMOS (Neuromyelitis optica Study Group). We requested data regarding clinical symptoms, antibody status, therapy regimen and response via a standardized questionnaire.

**Results:** During the 2-year recruitment period, 46 (both incidental and prevalent) patients with a suspected diagnosis of NMOSD were brought to our attention. Twenty-two of these patients did not fulfill the inclusion criteria. Of the remaining 24 children, 22 had a median age at onset of 11 (range 3–17) years and 16/22 were female (72.7%) (no data in two patients). Sixteen of 24 patients were AQP4-ab positive (67%), 4/24 MOG-ab positive (16.7%), three children were double-seronegative and in one patient no antibody testing was done. We calculated an incidence rate of 0.022 per 100,000 person-years for Germany, while there was no incidental case in Austria during the recruitment period. The prevalence rate was 0.147 and 0.267 per 100,000 persons in Germany and Austria, respectively.

**Conclusion:** Pediatric NMOSD, with and without associated antibodies, are very rare even considering the different limitations of our study. An unexpected finding was that a considerable proportion of patients was tested neither for AQP4- nor MOG-abs during diagnostic work-up, which should prompt to establish and disseminate appropriate guidelines.

## Introduction

Neuromyelitis optica spectrum disorders (NMOSD) are severe inflammatory demyelinating disorders of the central nervous system mainly characterized by simultaneous or sequential episodes of uni- or bilateral optic neuritis (ON), transverse myelitis (TM) and brainstem syndromes (BS) (1, 2). The discovery of a specific autoantibody in NMO patients, targeted against the water channel protein aquaporin 4 (AQP4) located in high density in astrocytic processes at the blood-brain barrier, in 2004 supported the differentiation between NMO and MS (3). In the following, multiple studies could show that AQP4-antibodies (AQP4-abs) are detectable in up to 80% of adult patients diagnosed with NMO (4–6). This led to a modification of the diagnostic criteria for NMO by adding the presence of AQP4-abs as a supportive criterion (7–10). Increasing AQP4-abs assay quality and reliability as well as further publications showing the connection between AQP4-abs and different clinical phenotypes other than ON and TM, resulted in another revision of the diagnostic criteria and led to the extension of NMO utilizing the umbrella term NMOSD (11–13).

Up to 20% of adult NMOSD patients remain AQP4-antibody (ab) negative (14–18) and a certain proportion of these AQP4-ab negative patients show antibodies against myelin oligodendrocyte glycoprotein (MOG-abs) (19–23). Recent studies showed that pediatric and adult NMOSD patients with MOG-abs can also have recurrent disease courses (24–28). The reported prevalence of MOG-abs in AQP4-ab negative pediatric patients shows a wide range between different working groups, possibly due to different inclusion criteria and unreported MOG-ab status. Consequently, in some studies the majority of NMOSD patients show MOG-abs while others report similar frequencies of AQP4-abs in pediatric as in adult patients (23, 29–32).

NMOSD is, by definition of WHO and EU, considered a rare disease with 1 (or fewer) in 2,000 individuals affected. Several population-based studies, focussing primarily on adult patients, showed incidence rates of 0.053 to 0.4 per 100,000 person-years and prevalence rates of 0.52 to 4.4 per 100,000 people (33–39). However, none of these studies applied the revised diagnostic criteria of 2015 and thus it remains unknown if (and how much) the incidence and prevalence rates were affected by the broadening of the spectrum. So far, Sepúlveda et al. 2015 criteria increased incidence and prevalence by 1.5 times (18). Hyun et al. even reported a 1.85-fold increase (40).

There are only a few studies focusing on the frequency of acquired demyelinating syndromes (ADS), among them NMOSD, in children (41–43). Very recently Boesen et al. specifically undertook a population-based, multicentre cohort study to estimate the incidence of pediatric NMOSD in Denmark, which was 0.031 per 100,000 person-years (44). An Australian single-center retrospective study could show that five of 67 (7.5%) pediatric patients presenting with ADS between 2007 and 2014 were diagnosed with NMOSD (43).

The aim of our study was to ascertain the incidence and prevalence of pediatric NMOSD in Germany and Austria, using the 2015 criteria. Subsequently, we evaluated if these patients would also fulfill the 2006 criteria and calculated the respective incidence and prevalence rates.

## Materials and Methods

### Setting

Germany and Austria are two geographically and politically defined countries located in Western and Middle Europe, respectively, with a combined area of 441,265 km^2^. Austria has 8,851,417 (30 October 2018 census) and Germany 83,019,213 (31 December 2018 census) inhabitants, resulting in a total of 91,870,630 people. By information of the Federal Offices of Statistics (Statistik Austria and Statistisches Bundesamt Deutschland) 1,535,958 and 13,597,428 respectively (combined 15,133,386), of these 91,870,630 inhabitants are underage (16.5%). Both countries have a majority of Caucasian ethnicity, however to our knowledge the official census institutions do not collect the population's ethnicities. Health care in Austria and Germany is provided by an open access public health care system with a network of pediatric neurologists specializing on demyelinating disorders.

### Case Ascertainment Tools and Study Populations

For this clinic- and questionnaire-based multicentre pro- and retrospective study we used the following tools to estimate the incidence and prevalence of pediatric NMOSD between 1 March 2017 and 28 February 2019:

1) We initiated a prospective epidemiological study via ESPED (Surveillance Unit for Rare Pediatric Disorders in Germany, in German “Erhebungseinheit für Seltene Pädiatrische Erkrankungen in Deutschland”; based in Dusseldorf) and included incidental cases of pediatric NMOSD diagnosed during the investigation period. Every pediatric department in Germany has one responsible colleague designated to report patients with newly-diagnosed rare diseases to the different ongoing ESPED studies.

2) Furthermore, we used ESNEK (Surveillance for Rare Neurological Disorders during Childhood, in German “Erhebung seltener neurologischer Erkrankungen im Kindesalter”; based in Göttingen) as an e-mail-based recruitment tool once a year during our recruitment period. Via ESNEK, we contacted all ~1,200 members of the Society for Neuropediatrics (Gesellschaft für Neuropädiatrie) and by this the majority of all Germany- and Austria-based pediatric neurologists and asked to report pediatric NMOSD patients, diagnosed and/or under their care during the recruitment period.

3) By using the e-mail distribution list of the pediatric neurology working group within the Austrian Society of Pediatrics and Adolescent Medicine, we contacted about 170 (and thereby most) Austria-based pediatric neurologists and asked to support our epidemiological study by referring pediatric NMOSD patients using our standardized questionnaire. As the majority of these colleagues are also part of the Society for Neuropediatrics, they received our e-mails twice each time.

4) Additionally, we included all pediatric NMOSD patients who were referred to our BIOMARKER study (based in Innsbruck and Datteln) between March 2017 and February 2019. This is an ongoing prospective, multicentre study started in 2009 with currently more than 900 included children and teenagers presenting with the first event of an ADS. We also included all previously referred children with ongoing follow-ups who fulfilled the inclusion criteria and were still <18 years old.

5) Finally, we contacted the adult NMO Study Group, called NEMOS, currently with representatives in 45 neurology clinics in Germany and asked how many pediatric (both incidental and prevalent) NMOSD patients had been brought to their attention between March 2017 and February 2019 and had been included in their patient registry. Representatives from about 25 German universities founded NEMOS in 2008 to improve the care of NMOSD patients (45).

### Study Population and Diagnostic Criteria

Patients included in this study had to meet the following inclusion criteria: (1) diagnosis of NMOSD fulfilling the 2015 International Panel for NMO Diagnosis criteria (12), (2) age below 18 years at disease onset, and (3) written informed consent. Exclusion criteria included the diagnosis of another type of ADS like MS or an infectious, metabolic, vascular, or neoplastic CNS disease.

### Standard Protocol Approvals, Registrations, and Patient Consents

The study was approved by the Ethics Committee of the Medical University of Innsbruck, Austria (Study number AN4059) and by the Ethics Committee of the Witten/Herdecke University, Germany (Study number 10/2017). All patients and/or their caregivers provided written informed consent.

### Antibody Assays

Serum samples were analyzed for the presence of MOG- and AQP4-abs by live cell-based immunofluorescence assays as previously described (46, 47).

Using the above-mentioned case ascertainment tools, not all serum samples were tested in Innsbruck. Serum of patients recruited via ESPED was not referred to our lab in Innsbruck and thus screened for MOG- and AQP4-abs by unknown assays. However, it is very likely that AQP4-ab testing was either done with a live cell-based assay (CBA) or with the well-evaluated Euroimmun kit (13). MOG-ab testing is possible with an Euroimmun kit as well, though the sensitivity and specificity are not as high as established live CBAs (47). Treating doctors might have sent serum samples to neuroimmunology labs in Heidelberg (S. Jarius) or in Kiel (F. Leypoldt), which both use the above-mentioned live CBAs.

### Statistical Methods

All of the 95% confidence intervals (95% CI) were calculated for the prevalence and incidence rate estimates using the modified Wald method. Quantitative variables were described using median and range. Statistical analysis was performed using IBM SPSS, release V.24.0 (IBM Corporation).

## Results

**Figure 1:**
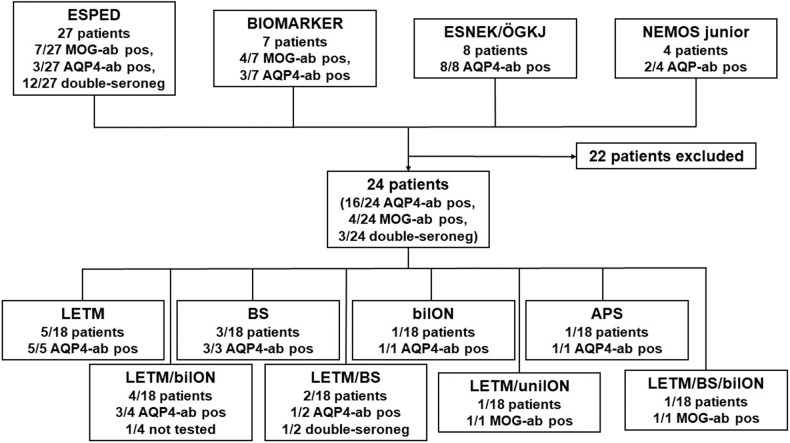
Flow chart study profile. APS, area postrema syndrome; AQP4-ab, antibodies against aquaporin 4; bilON, bilateral optic neuritis; BS, brainstem syndromes, ESNEK, Surveillance for Rare Neurological Disorders during Childhood; ESPED, Surveillance Unit for Rare Paediatric Disorders in Germany; LETM, longitudinally extensive transverse myelitis; MOG-ab, antibodies against myelin oligodendrocyte glycoprotein; ÖGKJ, Austrian Society of Paediatrics and Adolescent Medicine; unilON, unilateral optic neuritis.

With the above-mentioned ascertainment tools, a total of 46 pediatric patients with a suspected diagnosis of (both incidental and prevalent) NMOSD were brought to our attention. In 40/46 children we had enough clinical information necessary to evaluate the patients' diagnoses using the 2015 criteria. We identified 22 patients who did not fulfill the inclusion criteria and were thus excluded from this study (see Figure 1). None of the excluded patients fulfilled the 2006 criteria. All of these excluded patients were recruited via ESPED, meaning they were referred anonymously. Therefore, the type of antibody assay, used in 18/22 patients, was unknown. The remaining four patients were not tested for MOG- and AQP4-abs at all.

Of the remaining 24 children, 22 had a median age at onset of 11 (range 3–17) years and 16/22 were female (72.7%) (no data in two patients). In 18/24 patients, ethnicity was reported: 13/18 were Caucasian (72%), 3/18 (16.7%) were from the Near or Middle East or Egypt, 1/18 (6%) was African and 1/18 (6%) from South Asia.

Sixteen of 24 patients were AQP4-ab positive (67%), 4/24 MOG-ab positive (16.7%), three children were MOG- and AQP4-ab negative (double-seronegative) and in one patient no antibody testing was done. None of these patients were positive for both MOG- and AQP4-abs. In 19/23 patients, antibody testing was done with a live CBA. Thirteen of these 19 patients were screened in Innsbruck. The remaining four patients were recruited via ESPED and due to its anonymous referral method, we could not ask treating doctors which assay was used. However, as described above, it is very likely that well-established assays or kits were used.

Detailed clinical data was available for 18/24 patients (see Figure 1 and Supplementary Table 1). All 18 patients received intravenous methylprednisolone (IVMP) as acute therapy. Additional treatments at onset included intravenous immunoglobulins (IVIG; *n* = 7; 4/7 AQP4-ab pos, 1/7 MOG-ab pos, 1/7 double-seronegative, 1/7 not tested), plasma exchange (PLEX; *n* = 7; 5/7 AQP4-ab pos, 1/7 double-seronegative, 1/7 not tested) and rituximab (RTX; *n* = 2; 2/2 AQP4-ab pos). 14/18 pediatric patients were subsequently started on long-term treatments: RTX (*n* = 8; 1/8 after relapses on azathioprine; 7/8 AQP4-ab pos, 1/8 double-seronegative), azathioprine (AZA; *n* = 4; 4/4 AQP4-ab pos), tocilizumab (*n* = 2; after relapses on RTX; 2/2 AQP4-ab pos), mycophenolate mofetil (MMF; *n* = 1; not tested for autoantibodies), IVIG (*n* = 1) and cyclophosphamide (*n* = 1; after relapses on RTX; 1/1 AQP4-ab pos). We had no information about the type of DMT in one patient.

Of the remaining six patients without clinical information, four were referred via NEMOS with a confirmed diagnosis of NMOSD. Two of these four patients were AQP4-ab positive. The other two patients (not referred via NEMOS), both AQP4-ab positive, were brought to our attention as NMOSD without further details.

Further demographic and clinical details of these patients are summarized in Table 1 and Supplementary Table 1.

**Table 1:** Demographic and clinical characteristics of the included patients.

	**Patients (*n* = 24)**
Female sex, n (%)	16/22 (72.7%)
Ratio female:male	2.67:1
**Ethnicity, n (%)**	
Caucasian	13/18 (72%)
Near or Middle East or Egypt	3/18 (16.7%)
African	1/18 (6%)
South Asia	1/18 (6%)
**Antibody status, n (%)**	
AQP4-abs	16/24 (67%)
MOG-abs	4/24 (16.7%)
Double-seronegative	3/24 (12.5%)
Not tested	1/24 (4.2%)
Age at onset (years), median (range)	11 (3-17)
**Clinical attack at onset, n (%)**	
Longitudinally extensive transverse myelitis	5/18 (27.8%)
Bilateral optic neuritis + LETM	4/18 (22.2%)
Brainstem syndrome	3/18 (16.7%)
LETM + brainstem syndrome	2/18 (11.1%)
Bilateral optic neuritis	1/18 (5.6%)
Unilateral optic neuritis + LETM	1/18 (5.6%)
Area postrema syndrome	1/18 (5.6%)
Bilateral ON + LETM + BS	1/18 (5.6%)
Cerebral MRI, n (%)^*^	
Normal	5/18 (27.8%)
Non-specific WM lesions	6/18 (33.3%)
Brainstem involvement	5/18 (27.8%)
Optic nerves involvement	2/18 (11.1%)
Spinal MRI, n (%)^*^	
Normal	5/18 (27.8%)
LETM	13/18 (72.2%)
TM	0/18 (0%)
**Acute therapy, n (%)**	
Intravenous methylprednisolone	18/18 (100%)
Add-on therapy (IVIG, etc.)	8/18 (44.4%)
Long-term therapy, n (%)	14/18 (77.8%)

* *The minimum requirements to re-evaluate the referred MRI results were available imaging data with (contrast-enhanced) T1 and T2 for the spinal MRI and (contrast-enhanced) T1, T2, and FLAIR for the cerebral MRI. These criteria were fulfilled by 12/24 patients*.

### Study Populations

#### ESPED

During our observation period, a total of 27 pediatric patients were referred via ESPED with a diagnosis of NMOSD. However, 22 of these 27 patients (81.5%) with a median age at diagnosis of 14 (range 2–17) years, did not meet the diagnostic criteria (neither the 2006 nor the 2015): Seven of these 22 patients showed MOG-abs, 11 were tested negative for AQP4- and MOG-abs and in four no antibody testing was done. Clinically, 11 presented with ON, six with TM, three with LETM, and two with simultaneous TM (meaning less than three involved segments on spinal MRI) and ON. All these 22 pediatric patients were excluded.

The remaining five patients fulfilled the inclusion criteria. AQP4-abs were detected in 3/5 children (3 males, median age 7 [range 4–11] years), while one 13 year-old male patient was double-seronegative (patient 14) and in one 9 year-old female patient no antibody testing was done as she had already received IVIG prior to sample collection (patient 3). The cumulative median age was nine (range 4–13) years, 4/5 patients were male. Clinically, one AQP4-ab positive patient presented with an area postrema syndrome (patient 1), two with a simultaneous LETM and bilON (1 AQP4-ab positive patient and patient 3) and two with a simultaneous LETM and BS (1 AQP4-ab positive patient and patient 14).

Four of five patients did not only receive IVMP as acute treatment, but also at least one of the following: PLEX (*n* = 4), IVIG (*n* = 3) and RTX (*n* = 2). These four children were also put on long-term treatments: RTX (*n* = 3) and MMF (*n* = 1).

#### ESNEK and Pediatric Neurology Working Group Within the Austrian Society of Pediatrics and Adolescent Medicine

By contacting the majority of the Germany- and Austria-based pediatric neurologists via e-mail, seven so far unknown patients were brought to our attention. Another patient was already part of our BIOMARKER Study but reported again via ESNEK.

All these eight patients were AQP4-ab positive. Two patients were referred only stating their antibody status and without further demographic or clinical details. The remaining six patients had a median age of 13 (range 10–17) years and all of them were females. Interestingly, 5/6 patients initially presented with LETM. The remaining patient (patient 29) had a BS.

All patients were given IVMP, 3/6 additionally received IVIG and PLEX. Regarding the long-term treatment, we had no information about one patient (patient 39). Two of the remaining five children received the IL-6-receptor antagonist tocilizumab (after relapses on RTX), 1/5 azathioprine (AZA), 1/5 RTX and one patient did not respond to RTX and was changed to cyclophosphamide.

#### BIOMARKER Study

Since 2009, more than 900 children with a first (suspected) event of ADS were referred to our BIOMARKER Study and tested for MOG- and AQP4-abs. Within this cohort, seven patients fulfilled the diagnostic criteria and were still underage at the beginning of our observation period.

Four of seven patients were MOG-ab positive, 3/7 AQP4-ab positive. Their median age was 9 (range 3–14) years, 5/7 patients were female. One AQP4-ab positive, female patient (patient 33) had an ON as onset attack, the other two AQP4-ab positive children presented with a BS. The remaining four MOG-ab positive patients had simultaneous ON and LETM.

Every patient was treated with IVMP during the clinical event. Only one female, MOG-ab positive teenager (patient 43) additionally received IVIG. The three AQP4-ab positive patients were started on AZA and one was changed to RTX due to insufficient therapy response. Among the MOG-ab positive patients, only one received IVIG as long-term treatment (patient 44).

#### NEMOS

By contacting NEMOS and asking to report NMOSD patients who were underage at the beginning of our observation period, we could include four additional patients into this study, who have not been identified with one of the other tools.

Two of these patients were AQP4-ab positive and the other two AQP4-ab negative. In the latter two MOG-ab status was unknown. The median age was 11 (range 6–16) years, all four patients were females. One AQP4-ab negative patient (patient 34) was newly-diagnosed during our observation period and was added to the incidental cases. Besides age, sex and antibody status we did not receive any further demographic or clinical details.

### Incidence and Prevalence

Overall, six Germany-based patients were newly-diagnosed with NMOSD during our observation period and thus considered as incidental cases. Within our 2-year recruitment period, no child was newly-diagnosed with NMOSD in Austria. The remaining 18 children had already been diagnosed prior to our recruitment period and were therefore categorized as prevalent cases.

Considering a total of 13,597,428 minors in Germany, we estimated an incidence rate of 0.022 (95% CI 0.005–0.066) per 100,000 person-years. If we included only the three AQP4-ab positive patients, the estimated incidence rate was 0.011 (95% CI 0.002–0.033) per 100,000 person-years. For Austria, lacking an incidental case, we could not calculate the incidence rate.

With 20 pediatric NMOSD patients, the estimated prevalence rate in Germany was 0.147 (95% CI 0.096–0.217) per 100,000 persons, considering only the 12 AQP4-ab positive patients, it was 0.088 per 100,000 (95% CI 0.0456–0.154). Double-seronegative pediatric NMOSD (*n* = 3) had a prevalence of 0.022 (95% CI 0.005–0.066) per 100,000, and the four MOG-ab positive patients, fulfilling the diagnostic criteria for NMOSD, 0.029 (95% CI 0.009–0.076) per 100,000. One of these 20 patients was not tested for MOG- or AQP4-abs (patient 3).

In Austria, considering a total of 1,535,958 minors and four reported pediatric AQP4-ab positive NMOSD patients, the prevalence rate was 0.267 (95% CI 0.105 to 0.524) per 100,000 persons.

If we apply the 2006 criteria, only four of the above mentioned, Germany-based six patients would count as incidental cases, with one of them being AQP4-ab positive. Respectively, the incidence rate would be 0.015 (95% CI 0.001 to 0.055) cases per 100,000 person-years, resulting in an ~1.5-fold increase if the 2015 criteria are used.

The prevalence rates would decrease to 0.118 (95% CI 0.073–0.184) per 100,000 persons in Germany and to 0.200 (95% CI 0.064–0.460) per 100,000 persons in Austria.

## Discussion

Using different case ascertainment tools to assess and calculate the incidence and prevalence rate of pediatric NMOSD in Germany and Austria, we detected only 24 pediatric patients who fulfilled the 2015 criteria of NMOSD during our 2-year observation period. Nevertheless, the estimated incidence rate of 0.022 per 100,000 person-years (95% CI: 0.0081–0.048) is comparable to the systematic registry-based incidence rate of 0.031 per 100,000 person-years (95% CI: 0.011–0.082) calculated in the study by Boesen et al. (44) with both confidence intervals overlapping.

We could further show that a significant number of referred cases did not fulfill the 2015 NMOSD criteria, indicating that pediatric neurologists without special expertise in pediatric neuroimmunology are not familiar with the recently revised criteria.

In total, 22/27 pediatric patients (81.5%) referred via ESPED were incorrectly classified as NMOSD. Our ESPED inquiry also revealed that in five patients (4/5 were excluded from this study) no antibody testing was done at all. As antibody status could have had important diagnostic and therapeutic implications considering the disabling potential of relapses in AQP4-ab positive NMOSD, we strongly encourage pediatric neurologists to screen for MOG- and AQP4-abs in pediatric patients with ADS (32, 48). Furthermore, this lack of information should lead to the creation of better guidelines, facilitating diagnosis and therapy of pediatric ADS patients, which should be disseminated among physicians caring for children in particular with NMOSD.

Theoretically, it could have been possible that treating doctors referred their patients to us not only via ESPED, but also via ESNEK. As ESPED patients are reported anonymously, there is a chance that we counted double-referred patients twice and by this, created a falsely increased prevalence rate. However, postcodes of all ESPED reported patients were available and were double-checked with the patients referred via ESNEK or the BIOMARKER Study. The same issue arose with the four patients who were included via NEMOS. For these patients, we compared available data (age and antibody status) with the remaining patients and could thereby exclude that they had already been brought to our attention by another ascertainment tool.

Using both the 2006 and 2015 criteria, we could demonstrate that implementing the 2015 criteria has increased the incidence rate in Germany by 1.5 times. Similar increases of the incidence rate were also shown in recent studies [1.5 times in Sepulveda et al. (18), 1.85 times in Hyun et al. (40)].

Another important issue is the heterogeneity of NMOSD in the subgroup of AQP4-ab negative patients either harboring MOG-abs or being double-seronegative, which we included both in this epidemiological study. However, considering for example the different pathogenic mechanisms in AQP4-ab positive NMOSD (astrocytopathy) and MOG-ab associated disease (MOG-AD; oligodendrocytopathy), we calculated the prevalence both for AQP4- and MOG-ab positive patients separately fulfilling the 2015 criteria for NMOSD (0.088 vs. 0.029 per 100,000 persons, respectively). Appreciating the pathophysiological, clinical and radiological differences between AQP4-ab positive NMOSD and MOG-AD (also MOG-ab disease, MOG-encephalomyelitis, MOG spectrum disorders) it seems reasonable in the future to not include MOG-ab positive patients with a clinical phenotype of NMOSD, but to consider them as a separate disease entity (17, 20, 21, 25, 28, 49–56). While the pathophysiology of the double-seronegative NMOSD patients is still not understood, the clinical management remains the same, so we kept these patients in our calculation.

Sixteen of 24 (67%) pediatric patients showed AQP4-abs, while only four patients had MOG-abs. These results are partly supported by the literature (29–31, 57–59). An explanation for the large proportion of AQP4-ab positive patients in our study might be the fact that awareness among physicians for NMOSD is especially high when their patients are tested positive for AQP4-abs. Still, children clinically presenting with ADS rather show MOG- than AQP4-abs (60). However, more than half of all MOG-ab positive patients remain monophasic and are thus unlikely to fulfill the 2015 diagnostic criteria for NMOSD (27, 61). MOG-ab positive patients fulfilling these criteria may not have been referred to our study due to the treating physicians' decision to classify these patients as MOG-AD.

Our study has the following strengths: (1) first study addressing the epidemiology of pediatric NMOSD in Germany and Austria, (2) usage of multiple ascertainment tools, and (3) available clinical data for 18/24 patients (despite focussing on epidemiological data).

However, several limitations need to be addressed: Specific registries, using medical data provided by insurances or the health care system, for pediatric NMOSD patients exist neither in Germany nor Austria. Accordingly, population-based studies are currently not possible in these countries. However, by using various tools to recruit patients, we tried to compensate for this limitation as much as possible.

We are aware of the fact that this study still has a certain selection bias as the referring colleagues do not represent the majority of all pediatric neurologists in Germany or Austria. For example, there is not a single patient referred from Berlin- or Hamburg-based tertiary care children's hospitals, which is very unlikely considering that these are the two biggest cities in Germany. Therefore, we assume that there is a certain proportion of pediatric patients with NMOSD who were not reported to one of our ascertainment tools.

Another limitation is that we are not aware of the type of assay used in four patients referred via ESPED and in 18/22 excluded patients. However, it is very likely that well-established live CBA or commercially available Euroimmun kits were used to screen for MOG- and AQP4-abs.

In 6/24 included patients, we did not have sufficient clinical data to verify the referral diagnosis NMOSD. However, 4/6 patients were AQP4-ab positive making the diagnosis rather easy, and the two AQP4-ab negative patients were referred by an NMOSD expert consortium convincing us of the diagnosis.

## Conclusion

Pediatric NMOSD, both with and without associated antibodies, are very rare disease entities. An unexpected finding was that a considerable proportion of patients was tested neither for AQP4- nor MOG-abs during diagnostic work-up, which should prompt to create and disseminate commonly available and easy-to-follow guidelines. Finally, we are convinced that multicentric studies with higher patient numbers are needed to evaluate the true epidemiology, long-term outcome and prognosis of pediatric patients with AQP4-ab positive and double-seronegative NMOSD as well as MOG-AD.

## Data Availability Statement

All datasets generated for this study are included in the article/Supplementary Material.

## Ethics Statement

The studies involving human participants were reviewed and approved by Ethics Committee of the Medical University of Innsbruck, Austria Ethics Committee of the Witten/Herdecke University, Germany. Written informed consent to participate in this study was provided by the participants' legal guardian/next of kin. Written informed consent was obtained from the minor(s)' legal guardian/next of kin for the publication of any potentially identifiable images or data included in this article.

## Author Contributions

CL and KR created concept and design of the study, jointly acquired, and analyzed the data. CL drafted the manuscript. MR supported the data analysis. MBr, E-MW, MBa, BK, KS, and MR acquired the data and critically reviewed the manuscript.

## Conflict of Interest

MBr, EW, KS, and MBa report no disclosures related to the present work. KR received speakers' honoraria from Novartis and served on the advisory board of the PARADIGM study. MR receives payments for antibody assays (MOG, AQP4, and other autoantibodies) and for MOG and AQP4 antibody validation experiments organized by Euroimmun (Lübeck, Germany). CL served as a consultant for Roche. BK received speakers' honoraria and travel support and served as advisor for Biogen, Celgene, Merck, Novartis, Sanofi-Genzyme, Roche, and TEVA. The handling editor declared a past co-authorship with one of the authors, MR.

## Supplementary Material

Supplementary Table 1 Demographic, serologic, clinical, radiologic and therapeutic characteristics of the included patients.

## Abbreviations

ab: antibody
abs: antibodies
ADS: acquired demyelinating syndrome
APS: area postrema syndrome
AQP4-abs: antibodies against aquaporin 4
AZA: azathioprine
bilON: bilateral optic neuritis
BS: brainstem syndromes
DMF: dimethyl fumarate
DMT: disease-modifying therapy
ESNEK: Rare paediatric neurological disease registry Germany
ESPED: Surveillance Unit for Rare Paediatric Disorders in Germany
GLAT: glatiramer acetate
IVIG: intravenous immunoglobulins
IVMP: intravenous methylprednisolone
LETM: longitudinally extensive transverse myelitis
MMF: mycophenolate mofetil
MOG-ab: antibodies against myelin oligodendrocyte glycoprotein
NMOSD: neuromyelitis optica spectrum disorders
OCB: oligoclonal bands
ÖGKJ: Austrian Society of Paediatrics and Adolescent Medicine
ON: optic neuritis
PLEX: plasma exchange
pos: positive
RTX: rituximab
TM: transverse myelitis
unilON: unilateral optic neuritis.
